# Harvard Odontological Society

**Published:** 1895-02

**Authors:** 

**Affiliations:** Harvard Odontological Society


### HARVARD ODONTOLOGICAL SOCIETY.

At the regular monthly meeting of the Society, held July 7, 1894, President Eddy in the chair, the following paper was read:

### THE CARE OF CHILDREN'S TEETH.

BY N. A. STANLEY, D.M.D., NEW BEDFORD, MASS.

Mr. President and Gentlemen,—The subject to which I wish to invite your attention this evening, and one which, I think, does not receive sufficient consideration among general practitioners, is the care of deciduous teeth.

The task of filling children's teeth is a very difficult one, and because of this difficulty, and the short period that the deciduous teeth are to be retained, they are often entirely neglected, or not given due consideration.

Preserving the first teeth is a very important duty, and one that every dentist should not shrink from, but make every possible effort to perform.

It is Nature's design that these teeth be kept in their position until the permanent teeth which follow them are erupted, and any deviation from this plan is detrimental to her purpose, which cannot be improved upon. It is pre-eminently the office of the dentist to assist Nature over this difficult period, and in the performance of this duty he will often find his resources taxed to the utmost.

What are some of the results that are likely to follow the too early extraction of the first teeth?

1. The jaw does not develop as it should and as it otherwise would.

2. The permanent teeth erupt more irregularly.

3. The permanent teeth that are erupted one or two years before they should be are not fully developed, and will not resist decay with the same vigor as teeth coming at their natural period.

What are some of the results which follow preserving the first teeth?

1. A good surface has been preserved so that proper mastication can take place, and the development of the jaw can go on to the extent Nature had intended.

2. The permanent teeth, having been kept back until their natural time of eruption, are now in a normal condition.

But this is not all that has been accomplished. The young patients have been taught the importance of taking care of their teeth, which lesson will, in all probability, last them through life. We are able to restore the natural contour to a decayed tooth by means of the various filling-materials at hand; but we should try to do more. We should so strive to do our work and instruct our patients that future generations may inherit a dental structure less prone to disease.

The time to begin the improvement of dental anatomy is in childhood,—fœtal life, if you please.

We should prescribe the lime needed to supply the demand upon the parent system during pregnancy. I have done this in my own practice with confidence in its efficacy, and always with the cordial approval of my patient. The lime supplied will aid in the development of the fœtus and thus preserve the structure of the mother's teeth.

No doubt every one of us can call to mind instances where mothers have through ignorance neglected this provision during such periods, and we have seen disastrous results follow. Nor should we cease the supply after birth, for the demand is then greater. Every dentist can recommend the use of lime-water in the child's food, and I believe some of the best results can be obtained by following this practice a few years.

Every practitioner has methods of his own, and if he is a wise man he will have occasion to change them very often. It is impossible to follow any fixed rule; the operator's judgment must counsel him in almost every case. In our professional relations with children the first thing to be obtained is their confidence, and in order to accomplish this we must be very patient and avoid hurting them all we possibly can. I never keep a very young child in the chair longer than ten or fifteen minutes for their first experience; if they become restless and uneasy it is better to dismiss them and finish the operation some other day. By this means I soon have the confidence and friendship of my little patients firmly established.

It is my custom to see them once in three months. I don't expect to always make a thorough excavation; it is impossible.

In filling approximal cavities in the front teeth I almost invariably use cement; in the greater per cent. of cases I think it more satisfactory than any other filling.

For crown cavities that are not too deep I use amalgam, but if they are badly decayed I prefer cement or gutta-percha, as a large amalgam filling is more apt to destroy life.

For buccal cavities and those at the margin of the gums I believe there is nothing better than gutta percha.

I have had greater difficulty with approximal cavities in the molars than any other; the corona-distal surface in the first molars, especially the lower jaw. The filling I have the best success with here is a combination of amalgam and cement, amalgam at the cervical wall. Should a pulp become exposed, I should at once proceed to destroy it, and fill the pulp-cavity with cotton and iodoform.

Finally, we have to extract these teeth. The agent I have used for a number of years is chloride of ethyl, which works beautifully; it is unnecessary to spray the parts until white; this might cause sloughing.

The children of the present generation should have, when they grow up, better teeth than their fathers and mothers have to-day. Without careful, faithful attention to the first teeth, I believe it impossible to attain results which our patients have a right to expect.

DISCUSSION.

*Dr. Noble.*—I would like to ask Dr. Stanley what preparation he uses to destroy the pulps of temporary teeth?

*Dr. Stanley.*—Arsenic.

*Dr. Noble.*—Might not complications arise from the use of arsenic where the roots are partly absorbed?

*Dr. Stanley.*—I will not say that there might not, but I have never seen a case. I should use a *very small* particle and see the patient within a few hours; the same day, if possible.

*Dr. Cooke.*—Will Dr. Stanley please tell us how he knows that these permanent teeth that erupt early do not resist decay as well as they would if they erupted two or three years later?

*Dr. Stanley.*—From observation.

*Dr. Cooke.*—Do you compare it in the same mouth, or compare one mouth with another?

*Dr. Stanley.*—In the same mouth.

*Dr. Cooke.*—What teeth do you notice go quicker on that account?

*Dr. Stanley.*—I think the bicuspids will suffer most.

*Dr. Cooke.*—What sort of cavities do you find in such a case, fissure or approximal?

*Dr. Stanley.*—Fissure.

*Dr. Cooke.*—I don't know whether a case of that kind would prove that the tooth would not last through life if proper attention was given to the cavities. Whether the early eruption would have any influence against the ultimate keeping of the tooth through life it seems to me is a question.

One other point occurred to me in connection with the deciduous teeth, and I would like to ask the essayist or any of the gentlemen here whether trouble has been experienced with the six front teeth, either above or below? For instance, we have a great deal of toothache in the temporary molars, and we see in the incisors above good-sized cavities, and in many of them the pulps are dead. I have been running the matter over in my mind, and I cannot recall a case that has caused me any trouble in treating a tooth or has required any particular attention in any of the six front teeth. Our work is always in the molars, and if there are any cavities in the incisors they will not cause the patient much, if any, trouble, because there is not much pressure there, while the constant crowding in of food between molars and bicuspids is what brings the patient to us. I have noticed cavities in the incisors, but I have never tried to fill them, because they did not seem to require attention. I should like to know if the others present have had the same experience in this matter.

*Dr. Stanley.*—In the majority of cases the trouble proceeds from the molars, but I have had cases where the canine caused a great deal of trouble. I think there have been two occasions where I have had to treat the canine, which caused much pain and swelling, and in another instance there was similar trouble with the left superior central.

*Dr. Cooke.*—My question was, Does trouble come as a rule from the six front teeth?

*Dr. Stanley.*—I should say not.

*Dr. Reilly.*—In answer to Dr. Cooke's question, I would say that I have considerable trouble with the temporary canines, almost as much as with the temporary molars. I had one case in my own family, my little boy. Decay had started in the canines, and I was obliged to remove the fillings, and have an assortment of instruments at home to keep the canals clear. I have had many patients in my practice where there was trouble with the canines. With the centrals I don't recall any serious trouble.

*Dr. Stanley.*—In the particular case I referred to the crown finally broke off, but I intend to keep the root there if possible until there is some sign of the permanent tooth making its appearance. I treated the root, leaving a dressing of cotton and iodoform, and then filled with cement, and left a small opening through the filling. It has been there for some weeks, and has not given any trouble. I shall not be surprised if it does, but I shall treat it again in the same way.

*Dr. Cooke.*—I had in mind incisors more than canines.

*Dr. Smith.*—I would like to ask the essayist his method of treating the temporary molars after he has destroyed the pulp?

*Dr. Stanley.*—I should syringe the tooth out with warm water and treat with a three-per-cent. solution of pyrozone; then I should apply a dressing of cotton and iodoform to the pulp cavity, and put my filling over that immediately, keeping it as dry as possible while I work. I should not undertake to fill the roots solidly.

*Dr. Smith.*—Do you use the powdered iodoform?

*Dr. Stanley.*—No, I make a paste, and saturate the cotton with it. Of course you cannot do an ideal operation on a child five years old; you have simply to do the best you can.

*Dr. Page.*—My youngest patient with abscess was a child two years and nine months old.

I have always given the same treatment to the deciduous teeth that I have to the permanent, and in the case of an exposure I use arsenic as I do for a grown person, except that I use a much smaller quantity. I have a large number of those exposed pulps and abscesses, and it is extremely rare that I have one to re-treat. I have always practised Moffat's treatment, which I was taught at the school,—that is, a thorough opening of the pulp-chamber into all the root-canals, treating with creosote and tannic acid. With this treatment I have succeeded better than with all others that I have tried.

*Dr. Reilly.*—Did I understand Dr. Page to say that he does not fill the roots of deciduous teeth?

*Dr. Page.*—I do fill them.

*Dr. Stanley.*—I do not want Dr. Smith to think that I do not treat the permanent teeth more than once. It is about ten years ago that he taught me how to treat a tooth in which the pulp had been destroyed, and it has remained by me ever since.

*Dr. Smith.*—I did not suppose that for a moment. I wanted to know how you treated the temporary teeth. The method of treating the canals by thoroughly disinfecting the pulp-chamber is one that has been pursued by Dr. Allan, of New York, with a great deal of success, and has been published in one of the dental journals; it consists in making it thoroughly aseptic, placing a small disk over the entrance into the pulp-chamber, and then filling the tooth. It seems to me a very good way, having the advantage of being doubly sure, for, should there be any trouble, it is a simple matter to drill a vent below the margin of the gum, and it would not be difficult to remove the entire filling.

I was interested in what Dr. Cooke said about the deciduous anterior teeth. It struck me forcibly, because his method of handling the incisors is evidently the same as I pursue,—that is, I don't handle them at all unless I am obliged to; in fact, it is rare in my practice that I find it necessary to fill the temporary incisors, either superior or inferior.

*Dr. Werner.*—Have you ever filled a temporary incisor?

*Dr. Smith.*—I don't remember of any instance where I have. My associate, Dr. Banfield, has a little boy, a patient of his, about five years old, for whom he is filling the temporary incisors. I know of the case simply from his telling me about it, and have not seen it. I am obliged to fill the temporary cuspids and temporary molars, and there my work with children's teeth seems to end. It seems important that we should have a satisfactory method of filling the approximal cavities in temporary teeth, and I shall be glad to hear from the members the different ways of treating these cavities. At one time I used to file these teeth apart and treat them on a bevel, carrying the separation down to the gum margin, leaving the teeth in contact, and then filling the approximal cavities on that bevel, but the food would get in more or less, and now I do not file between temporary teeth, neither do I separate with wedges, as some do, but my method of late has been to cut down from the crowns into both approximal cavities, and fill those solidly with gutta-percha, and it works beautifully. There is no trouble with gum tissue, and when it wears it can easily be replenished.

I rarely use amalgam in temporary teeth, as I am somewhat afraid of it. I have seen cases, little patients of mine, who have had the sixth-year molars filled with amalgam, and the pulps have died. A family, in which there are three little daughters, went abroad, and while there amalgam fillings were placed in the mouths of each of the children, and when they returned, there was a pulpless sixth-year molar in each of their mouths. I have seen evidence enough to satisfy me that the use of amalgam in temporary teeth oftentimes produces death to the pulp through its influence.

*President Eddy.*—May I ask Dr. Smith in what way it acts to cause the death of the pulp?

*Dr. Smith.*—I don't know what the influence is, but I have concluded it is a dangerous thing to use in soft teeth. I have seen many bad results from it.

*Dr. Werner.*—Mr. President, I would like to say a few words. Gutta-percha in approximal fillings of temporary teeth I spoke of some fourteen years ago. I do not claim that it was original with me, but I have practised it for perhaps eighteen years, and in nearly all cases of approximal cavities between the bicuspids and sixth-year molars I would treat them in that way.

I want to talk in the line of Dr. Cooke's questions, which he did not follow out as much as I hoped he would, whether the premature extraction of the temporary teeth in any way affects the eruption of the permanent. That is a very vital question, and I do not agree at all with the statement of the essayist in regard to it. I do not believe that the extraction of the deciduous teeth has anything to do with the eruption of the permanent. It could only make a difference of a very few weeks or a very few months, and could have but very little influence in regard to the character of the erupting tooth. When the essayist spoke of that I think he must have had in mind cases where the eruption of the bicuspids has varied from two to four years, when normally the second bicuspid should erupt the year following the first. To my mind that is not at all the effect of the extraction of the deciduous tooth, but rather the effect of a characteristic inheritance, and, I think, on inquiring into such cases you will find that their fathers, their mothers, or some relatives have had the same peculiarity, and you will often observe it in a good class of teeth.

The sensitiveness of the deciduous tooth is nothing compared with the permanent. In destroying the pulp I should use arsenic just as freely in the deciduous tooth as in the permanent. There would not be a particle of danger in allowing it to remain there four or ten days or weeks.

Amalgam should very seldom be used in a child's mouth. I do not see the necessity of putting it in for the short time it is required.

*Dr. C. M. Bailey.*—I want to emphasize what the gentleman has said about the influence of the extraction of the deciduous teeth upon the eruption of the permanent. The essayist stated that one objection to the extraction of temporary teeth was the influence of that action upon the growth of the jaw, and consequently the growth of the jaw was retarded. I know that is the teaching, but I wish to take exception to that; I question it very seriously, and on the same grounds exactly that Dr. Cooke questions a tendency to decay resulting from the too rapid eruption of the permanent following the extraction of the temporary. We find in all ages all sorts of irregularities, notwithstanding the care which may have been taken in the attempts to have the permanent teeth erupt in their regular line. It seems to me the work of building up the tissue is a physiological process which is bound to go on, and I doubt whether our interference will reverse the order of nature and cause a contraction rather than an expansion of the jaw. The causes of irregularities of the teeth I think are but very little known. I do not claim to be able to give any reasons for them, but the question is simply whether we have been making a mistake in that line in our teachings in the past and have forgotten that the alveolar process is a bone built around the roots of the teeth for the purpose of holding the teeth in. Of course, we must admit that in crowded cases the bone was too small, but the only thing I question is that the early extraction of the temporary teeth induced that irregularity or caused the contraction of the jaw.

In regard to the filling of the temporary teeth, I do not think I have anything to add to what has already been said, except to say to Dr. Page that he will get as good results if he does not attempt to fill the roots of deciduous teeth as he will if he does. One thing is certainly true with our little patients and their teeth,—they will not so quickly become the subject of disease as the permanent teeth, and ulcerations are not so persistent. Nature is in active operation building up, repairing the process of waste, and carrying away the waste, and it does it better than it does for an adult, so there is not so much danger of those results that we dread with adults, and in the matter of the sensitiveness of the pulp of a deciduous tooth my own experience has accorded with that of Dr. Werner. They are not as sensitive, they more readily yield to the palliative treatment, and give less pain, and more readily succumb to any attempt to destroy them than do the pulps in permanent teeth.

*Dr. Page.*—I have had many centrals and laterals to fill, and I think my records show that a good many of them were abscessed. In many cases, as near as I could make out, the cause of the pulpless condition was a blow or some injury.

*Dr. Reilly.*—I would like to ask Dr. Smith what he does with caries of the deciduous incisors, or if he has no dead pulps?

*Dr. Smith.*—I see decay there, but I keep watch of it, and my experience is, the child has no trouble whatever in the way of toothache, and the teeth stay there until their successors follow them.

*Dr. Reilly.*—Don't they keep on decaying?

*Dr. Smith.*—Probably they do; but if they serve their purpose as temporary teeth, why fill them and cause your patient unnecessary pain and expense? I think that is a matter regarding which there should be a definite course of action. Take, for instance, a young man just from dental college: he would be apt, when the little child came to his office, to think that it was his duty to fill those teeth, but if he would let those cases alone, I think he would find that they would not cause him or the child any trouble.

I would like to ask Dr. Bailey, if I understood him rightly in his statement that the premature extraction of temporary teeth in no-wise affects the condition of the permanent teeth, in regard to their irregularity?

*Dr. Bailey.*—What I meant to say was that it does not affect them by causing a contraction of the jaw, or by the arrest of the development of the jaw of the child.

*Dr. Smith.*—I would agree with Professor Bailey in that opinion. I think it is generally so understood. Dr. Tomes cites a case where all the temporary teeth were taken out and the permanent teeth came in their regular order of development and were in line. Now, I am not quite clear what the essayist said on this point, but if I am not mistaken he claimed that the extraction of the deciduous teeth produced irregularity, not so much in the change of the jaw itself as in allowing the permanent tooth to move forward and erupt in a wrong position.

*Dr. Stanley.*—The trouble comes in allowing the sixth-year molar to come forward into the second bicuspid's place, and then you get a crowded condition of the teeth; with the incisors and bicuspids in place there is no room for the cuspid to take its position; it is erupted outside the arch and a contracted jaw is the result. I think it should be emphasized that the deciduous teeth be kept in position until their successors appear.

*Dr. Smith.*—I think that follows if the temporary molar has been removed before the permanent molar has erupted.

*Professor Bailey.*—I think the same thing. I question the advancing of that sixth-year molar if it has become firmly fixed in its position before the temporary molars have been extracted. I cannot say that I have demonstrated this, but simply put it forth as a portion of my faith, and I think the grounds of our assertions in the past need to be pretty thoroughly looked at before they are finally accepted. I think you will find that the edentulous mouths of which we take impressions do not average much smaller in such cases, showing that the jaw itself was the proper size.

*Dr. Cooke.*—One other point some of you may have noticed: where the pulps in the temporary molars have died early, and a fistula is found, the second teeth come down sooner than they do on the other side where the teeth are sound.

Referring to the matter of the filling of the front temporary teeth, I would say that I have had cases where I have filled them. One, a patient who is seventeen or eighteen years of age, still retains her temporary lateral incisors, and when they began to decay I filled them, because I doubt if she will ever have the second ones.

*Dr. Clapp.*—I would like to speak of a case that was in my office to-day,—the sixth-year molars in the mouths of two sisters. The elder has been an invalid, and on the grinding surfaces of the sixth-year molars there is no enamel. When the child first came to me a year ago these teeth were so sensitive that they were worse than useless for masticating purposes; in fact, when the child used them in eating they caused her a good deal of suffering, and it was a question what to do. I decided to use nitrate of silver, which has been applied several times since the first sitting and with very beneficial results. Of course, the teeth have no more enamel on them than they had previously, but the extreme sensitiveness has disappeared. The younger sister, whom I saw to-day, I am sorry to say, is having just the same trouble with the sixth-year molars that she is now erupting, and I propose to treat them in the same way.

*Dr. Grant.*—Speaking of the use of nitrate of silver, I want to ask if any one has tried a method recommended, I think, by Dr. Peirce, of Philadelphia, of applying nitrate of silver with blotting-paper? There is no danger of using too much nitrate, as the blotting-paper takes it up and it can be put anywhere without getting it in the mouth. For applying to the bottom of sensitive cavities in temporary teeth it is one of the best things that I have tried.

*Dr. Cooke.*—Does it change the color much?

*Dr. Grant.*—It gives a little blue color. It saturates the tooth less than when applied in any other way.

*Dr. Clapp.*—Do you fill over the paper.

*Dr. Grant.*—No, simply apply it with the paper; then make a thin pad of asbestos, put it down in the bottom of the cavity, and fill in with phosphate over that. No matter how sensitive the tooth is, you get the very best results, the asbestos is just as inorganic as zinc phosphate.

*Dr. Reilly.*—I don't quite understand the statement that there is so little trouble with the temporary incisors that it is just as well to let them alone. I have noticed the commencement of decay in my own children, and I have had a good chance to observe. The case that Dr. Cooke has just cited emphasizes the necessity for the treatment of every case. I always urge parents to be persistent in cleansing them and keeping the stains off, for there is nothing more hideous to me than a set of dirty teeth either temporary or permanent. It seems to me Dr. Grant's suggestion of nitrate of silver and blotting-paper would be a capital one if the discoloration was not too noticeable.

*Dr. Cooke.*—I remember reading something which is right in line with what Dr. Clapp has spoken of, where teeth were very sensitive at the neck, so much so that even the touch of the lip caused pain, and in that case a weak solution of cocaine with a very small amount of arsenic had a good effect.

*Dr. Reilly.*—How long did he let it stay there?

*Dr. Cooke.*—I don't remember. I know that Dr. J. D. White used arsenic a good deal for the extraction of hard roots, and also for the benumbing of the sensitiveness, and rendering excavation painless. I remember reading his description of it, and certainly no man in our profession was better qualified to judge of correct methods.

*Dr. Werner.*—I saw to-day a case which was treated two years ago in Lucerne by the American dentist there in the same way. I noticed that the buccal surface of the left upper third molar was perfectly black, and I said to the patient, "Nitrate of silver has been used there, hasn't it?" He said, "No; arsenic." I simply took it for granted that he was mistaken, and that it must have been the nitrate of silver. He is an inveterate smoker, and his teeth are coated with black deposit from smoking, and I question very much whether it was arsenic after all.

*Dr. Grant.*—It is almost the universal custom in France to use arsenic as an obtundent. The French people won't stand anything in the line of pain, so the dentists there use it quite freely.

*Dr. Cooke.*—Does it turn the tooth dark?

*Dr. Grant.*—Very often it does.

*Dr. Cooke.*—Dr. Taft had a case where he used nitrate of silver, and then applied something taking off the stain. I remember his speaking about it, but forget what he used. Perhaps he remembers the case.

*Dr. C. H. Taft.*—I used iodine and a solution of salt and water, and then cyanide of potassium, with very satisfactory result.

In regard to this question of obtunding sensitive dentine at the necks of teeth, while it is a little aside from the subject of the paper, I want to speak of a method which it seems to me has been overlooked, and yet it is so simple that I thought every one must use it. I think I was taught it in the dental school. It is to first touch the surface at that point with a little pledget of cotton dipped in alcohol, then thoroughly dry with hot air, and afterwards touch the surface with caustic potash or the chloride of zinc, which renders it insensible to pain or the touch of an instrument.

*Dr. Reilly.*—Do you mean on the enamel?

*Dr. C. H. Taft.*—Right at the neck of the tooth where there has been no decay.

As regards the subject of the paper, I was interested in what Dr. Stanley has put before us. The paper seems to have been discussed entirely from a surgical stand-point. The treatment and care of children's teeth, it seems to me, is very similar to that of the care of permanent teeth in adults. In the last two years I have found myself looking at the treatment of all diseases of the teeth and the oral cavity, not only from the surgical side but from the therapeutic as well, and I take the ground that the dentist has a great deal to do in common with the physician in the care of children's teeth, and that it comes within our legitimate field as specialists in medicine to prescribe medicines for the *prevention* of decay not only in the deciduous but in the permanent teeth. Let us take cases, for example, where dental caries is an inherited condition, as is evidenced in scrofulous, rachitic, and syphilitic patients, where we notice at once an insufficiency of the elements that go to make up the bony tissues. I know that the treatment which the physician directs in all such cases is essentially and necessarily a constitutional one, and that there are drugs which will help not only to *prevent* the decay of the deciduous teeth, but of the permanent ones that follow, and which will help to supply those inorganic substances of which the system stands most in need. Such remedies as creosote, causticum, calcarea carbonica, and calcarea phosphoricum are given by homœopathic physicians to make teeth strong and to supply the inorganic substances that enter into them, and therefore I contend that it behooves us as dentists to make such a study of the materia medica as will enable us to prescribe such remedies intelligently, and help to prevent the necessity for surgical treatment. I would like to ask Dr. Stanley if it has ever occurred to him to consider the care of children's teeth from a constitutional stand-point, and, if so, what therapeutic treatment he may have prescribed.

*Dr. Stanley.*—I recommend the syrup of lactophosphate of lime, generally prescribed by physicians. I have in mind two little children; whose teeth are almost perfect, though they are yet quite young, one of them being four or five, and the other a year or two older; and I have no doubt this result has been reached by giving them regularly a spoonful of lime-water in their milk. Their parents, both father and mother, had very poor teeth.

In the matter of irregularities, perhaps Dr. Smith expressed exactly what I meant better than I did myself. With the best treatment and the greatest care, the permanent teeth will not always erupt as we desire.

*Dr. Werner.*—Some one referred to the Arthur method in the treatment of deciduous teeth. I hope no one here has been unfortunate enough to try it, for I think that is the one black chapter in the history of American dentistry.

*Dr. Grant.*—I would like to make one or two observations in partial reply to what Dr. Werner has said. I had the pleasure of meeting Dr. Arthur when he was here in 1871. He wanted a cast of a perfect set of teeth, and, as I happened to have that, he took a cast of my mouth. At that time he tried to induce me to have my teeth separated, claiming they were so close together that approximal cavities would surely appear within a few years. I told him that they were good then, and they would stay as they were until they commenced going on their own hook. I have always been just as well satisfied that I did not consent, for I am now forty-seven, and no cavities have as yet appeared. The home of that sort of practice is about Baltimore, where, it seems to me, there is more of it done than in any other city, but I want to say that in some cases I am sure it has arrested decay. I have seen mouths where it was beautifully done years before, and not a trace of decay on any of those polished surfaces. I was very much impressed with it, and at times have almost wished that I had the courage to do it in cases where teeth were so much predisposed to approximal decay. I do not condemn the Arthur system so much as some men do. I do not want to be understood as recommending its use, but there are teeth——

*Dr. Werner.*—That will stand the treatment.

*Dr. Grant.*—Put it that way if you will; the fact remains that I have seen cases where decay had started in the incisors, and where there was every evidence of a tendency to approximal decay. After the bicuspids were cut and filed in that way, they remained in perfect order for years afterwards.

*Dr. Werner.*—Your own good common sense prevented you trying to experiment in your case.

*Dr. Stanley.*—I think those cases are exceedingly rare where you can file off a large portion of nature's protection and have it stand. I have had occasion to see cases in my practice. Dr. Davis was one of those who adopted this method of Dr. Arthur's, and he was a man who, if he believed in a theory, was sure to put it into constant practice. I have seen many cases where bicuspid teeth particularly were lost because the protection which nature put on them had been removed.

*Dr. Grant.*—I made those observations really with a strong bias against the system, and yet with a desire to be perfectly fair and to give it due credit. It was not always practised as intended. Some who claimed to be disciples of Arthur filed the teeth to a point and took off the approximal surface of the bicuspids. That was not Arthur's idea at all. He cut off the approximal palatal surface and left the buccal surface almost intact, so that there was a free, open, V-shaped surface from the buccal to the palatal surface, instead of the saw-shaped tooth which some dentists produced.

Subject passed.

Henry L. Upham, D.M.D.,
*Editor Harvard Odontological Society.*

